# Endoscopic Ipsilateral Interhemispheric Approach for Middle-Third Falcine Meningioma: A Case Report and Literature Review

**DOI:** 10.3390/brainsci13071085

**Published:** 2023-07-18

**Authors:** Gang Zhang, Junwei Wang, Pan Wang, Nan Wu

**Affiliations:** 1 Department of Neurosurgery, Chongqing General Hospital, Chongqing 401147, China; zhanggang_zg@yeah.net (G.Z.);; 2 Graduate Institute, Chongqing Medical University, Chongqing 400016, China

**Keywords:** endoscopic surgery, falcine meningioma, middle-third, ipsilateral interhemispheric approach, case report

## Abstract

Middle-third falcine meningiomas (FMs) are usually hidden deep under the eloquent cortex and abundant bridging veins, which represent the main hindrances to surgical access. The endoscopic approach has the advantages of wide visualization and free visual axis without areas of visual blindness, which allows for the resection of FMs with good visualization in a narrow space, especially in deep operations. Here, we report a case of a middle-third FM treated using the endoscopic ipsilateral interhemispheric approach. A 55-year-old female who had suffered a headache for 6 months without other remarkable symptoms was diagnosed with middle-third FM combined with imaging evaluation. According to the imaging performance and anatomical features, we scheduled the endoscopic ipsilateral interhemispheric approach to access and remove the tumor. Consequently, gross total tumor resection was achieved without surgery-related complications. After the surgery, the patient had an uneventful recovery and was discharged with no neurological deficit. During the 24-month follow-up, the patient’s condition remained favorable, with no tumor recurrence. In our opinion, the endoscopic ipsilateral interhemispheric approach is a feasible surgical strategy for FMs, which deserves further exploration as a treatment option.

## 1. Introduction

Falcine meningiomas (FMs) originating from the cerebral falx comprise approximately 5–9% of all intracranial meningiomas [1,2]. According to their relationship to the coronal and herringbone sutures, FMs can be classified into anterior, middle, and posterior third FMs [2]. Middle-third FMs are usually hidden deep under the eloquent cortex and abundant bridging veins, which represent the main hindrances to surgical access [3,4]. Generally, the shortest trajectory is preferable for the removal of meningiomas [5]. The microsurgical ipsilateral interhemispheric approach is a surgical challenge for middle-third FMs owing to the potential of the excessive brain retraction required to visualize hidden lesions to result in damage to the eloquent cortex and bridging veins, especially in deep or visual blindness areas [6,7]. However, the endoscopic technique allows for the resection of tumors with better visibility in a smaller space than the microscopic technique, and its indications are rapidly increasing [8,9]. In this article, we describe our surgical experience of the resection of a giant middle-third FM through an ipsilateral interhemispheric approach using an endoscope. Our case shows that the endoscopic approach is a feasible surgical strategy for FMs that allows for surgical manipulation with good visualization in the deep part of the interhemispheric fissure and at the surface of the lateral compressed gyrus, which may reduce the damage of veins and arachnoids and thus deserves further exploration as a treatment option.

## 2. Case Description

### 2.1. History and Examination

**Figure 1:**
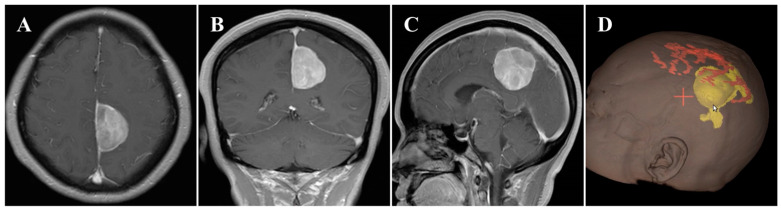
Preoperative imaging and intraoperative navigation showed a middle-third falcine meningioma. (**A**–**C**) Contrast-enhanced MRI axial (**A**), coronal (**B**), and sagittal (**C**) images. (**D**) Neuronavigation (3D image) demonstrated two drainage veins above the tumor (white arrow).

In November 2020, a 55-year-old female was admitted to our hospital and presented with a 6-month history of headache. On general physical examination, she presented with no other remarkable symptoms. MRI revealed a mass approximately 2.9 × 4.2 × 4.1 cm in size, with an attachment in the middle-third of the falx (Figure 1A–C). This parafalcine lesion was mainly extended to the left, with no significant perilesional edema. Neuronavigation showed two drainage veins above the tumor (Figure 1D), which is important for formulating the surgical strategy. Consequently, FM was diagnosed, and surgery was scheduled using the ipsilateral interhemispheric approach to access and remove the tumor using endoscopy through the safe zone between the two veins.

### 2.2. Surgical Treatment

**Figure 2:**
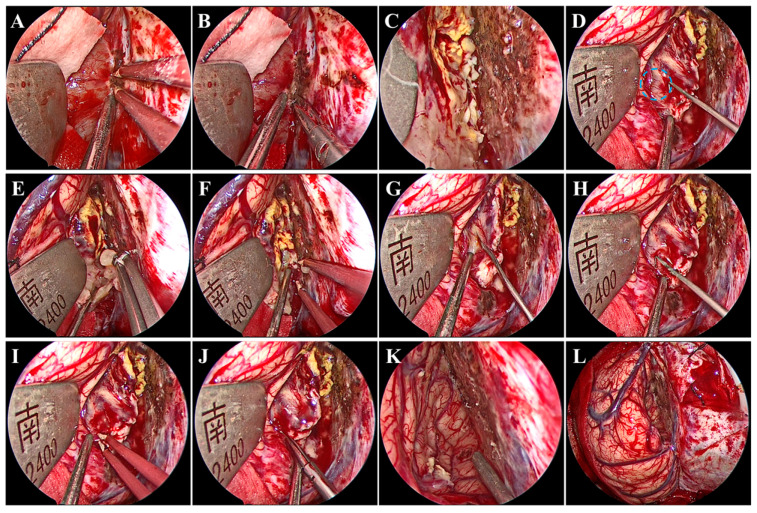
Intraoperative endoscopic views. Treatment of the tumor base with bipolar forceps (**A**) and microscissors (**B**). (**C**) Continued bleeding of the tumor. (**D**) Small anastomosing vessels (blue circle) between the drainage veins of the cerebral cortex and the tumor. Internal resection of the tumor using tumor-grasping forceps (**E**) and an aspirator (**F**). Dissection and separation of the tumor envelope using nerve strippers (**G**,**H**), bipolar forceps (**I**), and microscissors (**J**). Accurate hemostasis was verified (**K**) without damage to the bridging veins (**L**).

Under general anesthesia by endotracheal intubation, the patient was placed in the supine position. The head was fixed using a head clamp with no rotation. A horseshoe-shaped incision was made to expose the skull after identifying the location of appropriate craniotomy using neuronavigation. Next, a hole was drilled on the lateral edge of the superior sagittal sinus (SSS), and a 7 cm × 6 cm craniotomy was made. Particular caution should be taken to avoid damage to the SSS and bridging veins from dural laceration. After the removal of the bone flap, a radial incision was made on the dura mater with its base toward the SSS. Twenty minutes before the dura mater was opened, mannitol was administered to decrease the intracranial pressure. We introduced the endoscope following dural mater incision. A hand-held endoscope is often used to observe the tumor and develop a resection plan. A pneumatic arm holds the endoscope in one corner while the surgeon conducts bimanual surgery to remove the tumor. Subsequently, under endoscopic visualization, the arachnoid adhesions over the SSS were freed, while the bridging veins above the tumor were not. Notably, brain retraction was kept to a minimum. The falx was then separated to access the tumor in the depths. Once the tumor was visualized, electrocautery and dissection of the tumor base were performed using bipolar forceps and microscissors to block the blood supply from the falx (Figure 2A,B). After the treatment of the tumor base, the tumor was still bleeding (Figure 2C), which suggested that the tumor had an additional blood supply from the cerebral cortex (Figure 2D). Next, the tumor was internally resected in a piecemeal fashion with an aspirator and tumor-grasping forceps (Figure 2E,F). An adequately thick margin should be left to avoid damage to the cerebral cortex and to facilitate subsequent dissection of the tumor envelope. Subsequently, under endoscopic visualization, the tumor envelope was carefully dissected and separated from the surrounding cerebral cortex to remove the remaining tumor shell, all of which was performed with good visualization (Figure 2G–J). During this procedure, there was one area where the tumor tissue was tightly adherent to the cortical artery and extremely difficult to separate. Therefore, the tumor tissues around the artery were removed in a piecemeal manner and properly fulgurated, retaining a thin slice of the tumor (Figure 2K). Gross total tumor resection was ultimately achieved without damage to the bridging veins, and accurate hemostasis was verified under endoscopic visualization (Figure 2K,L). The major surgery process is presented in Video S1. Although it is easy to achieve a Simpson I resection after gross total tumor resection, in our procedure, instead of removing the cerebral falx at the base of the tumor, a fulguration was performed to preserve the falcine venous plexus (Simpson II). Finally, the dura mater was tightly sutured; the bone was secured using mini plates; and the skin incision was closed. The procedure took 253 min.

### 2.3. Postoperative Course

**Figure 3:**
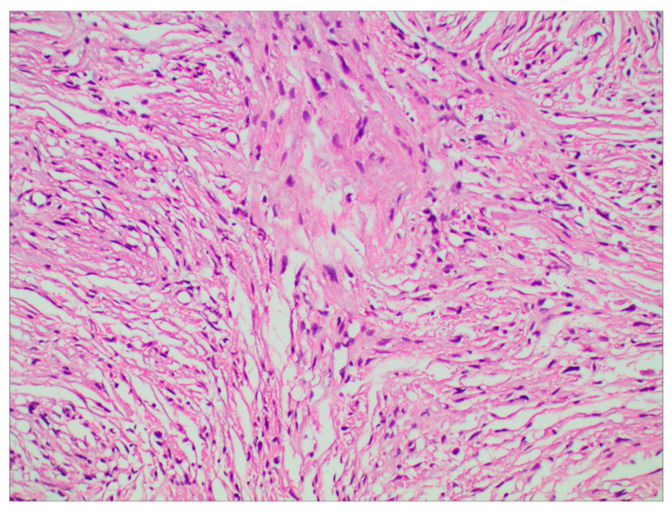
Photomicrographs of tumor specimens demonstrated a fibrous meningioma (H&E staining; original magnification ×200).

**Figure 4:**
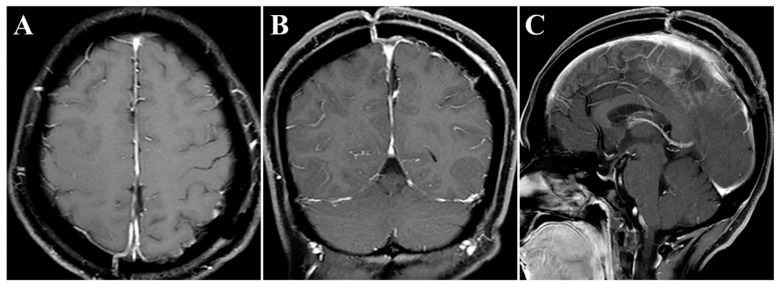
Postoperative contrast-enhanced MRI images. (**A**–**C**) Six months after surgery, MRI indicated regained parenchymal normal morphology. Axial (**A**), coronal (**B**), and sagittal (**C**) images.

The surgery was uneventful, and the pathological diagnosis was FM, WHO grade I (Figure 3). After surgery, the patient had an uneventful recovery and was discharged with no neurological deficit. The initial clinical follow-up was performed 6 months after surgery, at which point, MRI suggested that the compressed cerebral cortex had returned to its normal form (Figure 4). After 6 months of follow-up, subsequent follow-up visits were performed at yearly intervals or more frequently when indicated. During the 24-month follow-up, the patient’s condition remained favorable, with no tumor recurrence.

### 2.4. Literature Review

To review the cases of endoscopic resection of FM, the PubMed database (https://pubmed.ncbi.nlm.nih.gov/, accessed on 23 June 2023) was searched, and the available English literature meeting the set requirements was screened. The following terms were searched: (((“Meningioma”[Mesh]) OR (meningioma[Title/Abstract])) AND ((falcine[Title/Abstract]) OR (falx[Title/Abstract]))) AND (endoscop*[Title/Abstract]). Three irrelevant studies that did not report endoscopic resection of FM and two comments of a previous study were excluded. Based on the literature review (Table 1), three articles reporting six cases of FM resection using the endoscopic approach were identified between 2016 and 2022 [10,11,12]. One study [10] used the oblique surgical trajectory, and the other two [11,12] used the contralateral interhemispheric approach. Most of these cases achieved gross total resection without severe complications. The endoscopic approach obtained great efficacy.

## 3. Discussion

Resection of the middle-third FM poses a surgical challenge. Unlike the removal of convex meningiomas, middle-third FMs are usually hidden deep under the overlying eloquent cortex and abundant bridging veins; this positioning can lead to great difficulty in resection because impairment of these vital cortices and veins can cause serious surgical complications [13,14]. Indeed, injury to the precentral gyrus can lead to contralateral hemiparesis; damage to the postcentral gyrus can cause contralateral paresthesia; impairment to the paracentral lobule can manifest as contralateral lower limb weakness, sensory loss, or bladder incontinence; and disruption to the major bridging veins can result in venous infarction [2,15]. Therefore, in recent years, some authors have reported a contralateral interhemispheric approach, which can avoid edema aggravation of the ipsilateral cerebral cortex and ipsilateral bridging vein injury and provides a direct trajectory to the tumor base, allowing for easier cutting of most of the tumor blood supply [16,17,18]. However, using this approach may result in contralateral brain impingement with a longer surgical trajectory, causing potential damage to the normal cortices and veins on the contralateral side [6]. In addition, when the tumor is too close to the SSS, the contralateral approach is too difficult to implement. Moreover, the falcine venous plexus within the cerebral falx may function as a pathway in the intracranial venous circulation [19]. Additionally, the falcine venous plexus in the middle and posterior thirds are larger and denser than those in the anterior third, so careful consideration should be given to the resection of the cerebral falx, especially the middle and posterior thirds [20]. Therefore, in this case, the cerebral falx of the tumor base was treated by fulguration instead of being removed (Simpson II). Noteworthily, previous studies have demonstrated that the risk of recurrence is similar between Simpson grade I resection and grade II, and only higher (III or IV) resection grades were found to be associated with tumor progression [21,22,23]. Middle-third FMs are usually hidden deep, which leads to a tendency to perform conventional microscopic surgery to avoid damage to the eloquent cortex and bridging veins caused by the excessive brain retraction required to visualize hidden lesions. However, in our opinion, this can be prevented using an endoscope. The endoscope allows for a close-up view and obtains a good visualization even at a great depth, which facilitates safe tumor resection while avoiding damage to the eloquent cortex and bridging veins [10,11]. Additionally, the various angles of endoscopes (e.g., 0°, 30°, and 45°) can easily access the hidden lesions located in the blind spot of the microscopic approach [12]. To the best of our knowledge, there have only been three case series reporting endoscopic surgery for FMs, all of which reported small case samples (Table 1). In 2016, Spektor et al. [10] presented full endoscopic removal of an ant-third parasagittal meningioma using an oblique surgical trajectory. Their surgery was uneventful with no damage to the major veins, and subtotal resection of the tumor was achieved. Moreover, Roser et al. [11] reported a case where an ant-third FM was removed using an endoscopically assisted contralateral interhemispheric approach. In their study, the endoscope was used only in the final step of the procedure to verify hemostasis and locate tumor residues. In the article by Sakaeyama et al. [12], the authors described a series of four cases with FM (anterior, 2; middle, 1; and posterior, 1) treated by a full endoscopic contralateral interhemispheric approach. In all cases, the cerebral falx of the tumor base was removed to achieve Simpson grade I resection, regardless of the tumor location. Compared to the previous studies listed in Table 1, the major disparity of our study is the use of an endoscopic ipsilateral interhemispheric approach to remove the middle-third FM with the shortest trajectory, in which the cerebral falx of the tumor base was preserved. Another advantage is that the location of the bridging vein is not a hindrance to the endoscopic procedure [12]. Despite these advantages, the endoscopic approach is technically difficult and should be performed after sufficient experience with other simple endoscopic techniques. The long training process is the main limitation. In our department, we have performed more than 1000 endoscopic surgeries for the removal of pituitary neuroendocrinology tumors and a variety of skull base tumors, which was an important consideration for performing the current procedure. Additionally, this technique has the same adverse consequences as the microscopic approach, mainly including damage to the vital cortices and veins.

Noteworthily, in this case, there were two critical draining veins above the tumor, which increased the difficulty of tumor resection. However, we successfully removed the tumor using the endoscopic ipsilateral interhemispheric approach without any venous complications during or after surgery. Additionally, the presence of ipsilateral brain edema can narrow the entry corridor, representing a key concern of the ipsilateral interhemispheric approach. However, this concern can be overcome by using the gravity-assisted technique to gain more operating space, which is sufficient to allow safe and effective removal of the tumor using the endoscopic approach [6]. In this case, the main considerations for the use of the retractor were the presence of two critical draining veins above the tumor and the absence of severe ipsilateral brain edema. Gravity-assisted lateral positioning may increase tension in the veins, leading to injury, while the retractor is more controllable to keep brain retraction to a minimum. In other situations, such as the presence of severe ipsilateral brain edema or the absence of interference of draining veins, we would implement the gravity-assisted technique to extend the lateral corridor diameters. In the current case, postoperative MRIs showed that the overlying eloquent cortex returned to its original morphology (Figure 4).

In conclusion, our preliminary experience indicated that the endoscopic ipsilateral interhemispheric approach for FMs provided better intraoperative visualization, the shortest surgical trajectory, and early devascularization without the removal of the falcine venous channels. In addition to the merits listed above, several other factors should be noted. First, the endoscopic approach is technically difficult and requires a long training process. Second, the study is a case report with a limited number of cases, so the level of evidence is low. Therefore, future studies are warranted to confirm its effectiveness and safety. Moreover, we would continue to assess the safety and effectiveness of the endoscopic ipsilateral interhemispheric approach for FMs in our future work.

## 4. Conclusions

We share our surgical experience to remove a giant middle-third FM via an endoscopic ipsilateral interhemispheric approach. Our preliminary experience indicated that this approach deserves more consideration given the evident advantages compared to other approaches, including wide visualization and free visual axis without visual blindness areas, the shortest surgical trajectory without limitations of tumor depth, minimal brain retraction for the preservation of eloquent cortex and bridging veins, and early devascularization without removal of the falcine venous channels. Given the limited number of cases, future studies are warranted to confirm its effectiveness and safety.

## Figures and Tables

**Table 1:** Summary of literature reporting the use of an endoscope for falcine meningiomas.

Author/Year	Sample Size	Tumor Location	Surgical Approach	Extent of Resection
Spektor S et al. [10] 2016	1	Ant	Oblique surgical trajectory	STR; SG I
Roser F et al. [11] 2018	1	Ant	Contralateral interhemispheric approach	GTR; SG I
Sakaeyama Y et al. [12] 2022	4	Ant 2; Mid 1; Post 1	Contralateral interhemispheric approach	GTR; SG I
Zhang G et al. (present study)	1	Mid	Ipsilateral interhemispheric approach	GTR; SG II

Ant: Anterior, Mid: Middle, Post: Posterior, STR: Subtotal resection, GTR: Gross total resection, SG: Simpson grade.

## Data Availability

Data are available from the corresponding author upon reasonable request.

## Supplementary Materials

The following supporting information can be downloaded at: https://www.mdpi.com/article/10.3390/brainsci13071085/s1: Video S1: The major procedure of endoscopic surgery.
